# Commentary: Predicting blood concentration of tacrolimus in patients with autoimmune diseases using machine learning techniques based on real-world evidence

**DOI:** 10.3389/fphar.2022.1000476

**Published:** 2022-11-03

**Authors:** Yuxiang Zhu, Xuebin Wang, Xue Wang, Lizhi Chen, Zhuo Wang

**Affiliations:** ^1^ Shanghai Altimetria Cloudlink Healthcare Technology Co., Ltd., Shanghai, China; ^2^ Department of Pharmacy, Changhai Hospital Affiliated with Naval Medical University, Shanghai, China

**Keywords:** blood concentration prediction, tacrolimus, therapeutic drug monitoring, XGBoost, extreme gradient boosting, machine learning

## Introduction

This study investigated the predictive power of 14 machine learning models for the blood concentration of tacrolimus and found that the XGBoost model was the most accurate. However, we believe that the study has some problems in its research methodology, which may have skewed the results.

### Oversimplified feature selection

The researchers first used the XGBoost model to select the most important features from 52 candidates. Nine features were selected, and 14 models including XGBoost were trained with those features. It was found that XGBoost performed the best.

These nine features were selected simply because XGBoost performed well with them, so the fact that XGBoost performed best does not necessarily mean it is the most suitable for the task. It only shows that the feature selection process is slightly oversimplified.

One of the publications (Van Looy et al., 2007) cited in the article also used machine learning to predict tacrolimus concentrations with three models: linear SVR, RBF SVR, and MLR. It used each of the three models independently for feature selection and then trained the three models with the best set of features for each model. Linear SVR was found to be the best predictor, with RBF SVR being slightly worse, and both were much better than MLR. However, the linear SVR model used 15 features, MLR used 16, and RBF SVR required only 2. In addition, both linear SVR and MLR performed worse than RBF SVR when trained with the two features selected by RBF SVR.

This finding suggests that models work well only with suitable features. When only one model is used for feature selection, the results are likely to be favorable.

### Possible underestimation of models

We believe that the following problems may exist and could lead to an underestimation of the predictive power of the models.

1) It is possible that there was no hyperparameter tuning. Hyperparameter tuning is an essential process in all machine learning projects; however, the article does not mention whether it was performed. The default hyperparameters may have been used for each model. If the authors had tuned the hyperparameters, they should have reported the tuning results because it is a common practice to do so in the machine learning industry.
2) It is possible that there was no feature scaling. Many models are sensitive to data distribution. Therefore, feature scaling before feeding the data into the models is recommended. For example, KNN is highly sensitive to the range of features. Among the nine features selected, height had the largest median of 157, whereas some other features had a median of only 0. This means that height has a disproportionate impact on the output of KNN, and it will undoubtedly perform poorly if no feature scaling was performed. If the features were scaled, the scaling method used should also be reported.

According to Table 2, a prediction is considered correct when it is within 30% error of the actual value, then half of the 14 models have less than 55% accuracy, and the worst performing LASSO regression has only 48.7% accuracy.

However, if a model blindly outputs a tacrolimus concentration of 4.2 all the time, the predictions are correct when the actual values are between 3.23 and 6. This means that a meaningless model such as this has nearly 50% accuracy and possibly beats the LASSO regression, given that the IQR of tacrolimus concentration in the dataset is 3–6. This is likely due to the possible lack of hyperparameter tuning and feature scaling, as well as an oversimplified feature selection process.

### Questionable data

#### HCT

According to Table 1, the median of the feature HCT (hematocrit) is 0, and the IQR is 0–0 in the dataset. This means that more than 75% of the data had an HCT of 0, which is far from the normal value.

HCT was ranked as the third most important feature in this study. However, with more than 75% of the data having the same HCT value of zero, it is very unlikely that this feature is of high importance.

#### Height and weight

The units of height and weight are mislabeled kg and cm, respectively, in Table 1. Height is listed as the most important feature by XGBoost in this study, whereas weight normally has more impact in previous studies. Given that the surprisingly high influence of height is discussed in this article and is one of the major findings of this article, the mislabeling of units makes us wonder whether it is a clerical error or whether the weight feature in the dataset was somehow labeled height.

#### DBIL and some other features

The medians and IQR of TBIL, DBIL, IBIL, and plasma D-dimer in the dataset all depart from distributions observed in clinical practice, and DBIL was selected for model training.

### Clerical error

In the section titled Clinical Interpretation, the article states that the feature value in Figure 4 “means the contribution of each variable to the predictive power of the model.” However, this feature value indicates the original value for each variable. This could have been a clerical error.

### Room for improvement

Figure 3 shows the predicted values *versus* actual values as a scatter plot, from which it can be seen that all actual tacrolimus concentration values greater than 8 are underestimated by the model, forming a separate cluster in the plot.

Given that the dataset is small and unbalanced, with more than half of the data coming from people under 18 years of age, this consistent underestimation is most likely due to the lack of data on high tacrolimus concentrations. If it is not feasible to collect more data, potentially useful mitigation options include the following.

1) Data transformation. Log transformation or other transformations can be applied to tacrolimus concentration.
2) Performing analysis of covariance to evaluate the effect of the adult/child factor or only using data from children to train models.

## Conclusion

Machine learning has great potential in the healthcare industry, but we also need an in-depth understanding of the models and data processing techniques used instead of simply feeding data into a model.
